# Association between VEGF Gene Polymorphisms and the Susceptibility to Lung Cancer: An Updated Meta-Analysis

**DOI:** 10.1155/2018/9271215

**Published:** 2018-06-14

**Authors:** Fengming Yang, Zhiqiang Qin, Chuchu Shao, Weitao Liu, Ling Ma, Yongqian Shu, Hua Shen

**Affiliations:** ^1^Department of Oncology, The Affiliated Sir Run Run Hospital, Nanjing Medical University, Nanjing, Jiangsu Province, China; ^2^Department of Oncology, The First Affiliated Hospital of Nanjing Medical University, Nanjing, Jiangsu Province, China; ^3^Department of Urology, The First Affiliated Hospital of Nanjing Medical University, Nanjing, Jiangsu Province, China; ^4^Department of Pathology, Nanjing Medical University, Nanjing, Jiangsu Province, China

## Abstract

**Background and Objective:**

The association between vascular endothelial growth factor (VEGF) gene polymorphisms (-2578C/A, +936C/T, and -460C/T) and lung cancer risk has been extensively studied in the last decades, but currently available results remain controversial or ambiguous. Therefore, we conducted a meta-analysis to assess whether the relationship between the VEGF gene and lung cancer susceptibility exists.

**Methods:**

The meta-analysis was conducted by searching the databases PubMed, Embase, and Web of Science covering all eligible studies published up to October 1, 2017. The pooled odds ratios (ORs) as well as their 95% confidence intervals (CIs) were utilized to evaluate the possible associations. Publication bias of relevant studies was examined via Begg's funnel plots and Egger's regression tests.

**Results:**

This meta-analysis included 13 published case–control studies covering 4477 patients with lung cancer and 4346 healthy controls, who had been accrued from December 1992 to July 2012. For the overall eligible data collected in our meta-analysis, it indicated that VEGF +936C/T, -460C/T, and -2578C/A polymorphisms did not correlate with the elevated lung cancer risk in all genetic comparison models. Moreover, VEGF +460T/C polymorphism was found to be significantly associated with susceptibility to lung cancer in these models (allele model: pooled OR = 1.12, 95% CI: 1.00–1.26,* P* = 0.184; homozygote model: pooled OR = 1.51, 95% CI: 1.12–2.03,* P* = 0.821), but no significant results were detected in Caucasian populations.

**Conclusions:**

VEGF +936C/T, -460C/T, and -2578C/A polymorphisms were not associated with the risk of lung cancer. The VEGF +460T/C polymorphism might be a risk factor for lung cancer only in Asian populations.

## 1. Introduction

Lung cancer, as one of the most frequently occurring cancers in the world, is always the leading cause of cancer-related death among both men and women [1]. Approximately two million people are diagnosed with lung cancer each year and most of them diagnosed at an advanced stage [2]. The lack of effective treatment options and high mortality make lung cancer a major public health challenge all over the world [3, 4]. With the advent of next-generation genotyping and in-depth understanding of the molecular biology of lung cancer, genotyping of single-nucleotide polymorphisms (SNPs) may be pivotal in the personalized treatment for patients with lung cancer [5].

Angiogenesis refers to the complex process of the formation of new networks of blood vessels. Increased angiogenesis has been proved to be associated with the process of tumor growth and metastasis [6]. Vascular endothelial growth factor (VEGF), also known as vascular permeability factor, is one of the most vital regulators of angiogenesis and vascular permeability. It is well known that VEGF plays a critical role in the progress and prognosis of malignancy [7, 8], which has been confirmed* in vitro and in vivo *[9, 10]. Serum VEGF levels of cancer patients were significantly higher than those of healthy controls [11]. Moreover, serum VEGF levels always can predict the effects of chemoradiotherapy sensitivity in cancer patients [12]. In lung cancer, VEGF is important in establishing a vascular supply within the tumor [13]. The VEGF/VEGF-receptor axis is composed of multiple ligands and receptors with overlapping and distinct ligand–receptor binding specificities, cell-type expression, and function [14]. Targeted inhibition of the VEGF signaling pathway can partially suppress tumor angiogenesis and growth. In recent years, the use of anti-VEGF antibodies, such as bevacizumab, has shown a favorable clinical efficacy in treating non-small cell lung cancer (NSCLC), especially when used in combination with chemotherapy [15, 16].

The VEGF gene is located at chromosome 6p21.3, covering 14 kb in length with 8 exons and 7 introns [17]. Being highly polymorphic, at least 30 single SNPs have been identified and described [18]. Several SNPs of VEGF have been reported to be associated with individual susceptibility to cancer and can alter the VEGF expression and protein production [19, 20]. For example, −634G>C SNP in the 5′-UTR of VEGF affects the protein translation efficiency, and 936C>T SNP in the 3′-UTR influences the expression of VEGF in tumor tissues [21, 22]. SNPs, such as VEGF -2578C/A, +936C/T, and -460C/T, have been reported to be associated with cancer susceptibility, tumor growth, and radiotherapy sensitivity in patients with lung cancer [23, 24]. Recently, accumulating evidence has shown that VEGF aberrations may contribute to the development of lung cancer [25]. However, due to the limited sample size, the results of these studies remain controversial or inconclusive. In recent years, related studies were updated; in particular a study included in a previous meta-analysis was withdrawn [26]. Thus, this updated meta-analysis including all eligible case–control studies was performed to investigate whether VEGF polymorphisms were associated with the risk of lung cancer.

## 2. Materials and Methods

### 2.1. Search Strategy and Selection Criteria

A systematic literature search was conducted on PubMed, Embase, and Web of Science databases until October 1, 2017, to identify studies for inclusion in the meta-analysis. The relevant key words and search strategies were as follows: “vascular endothelial growth factor” or “VEGF,” “polymorphisms” or “mutation,” “variants” and “lung cancer,” “non-small cell lung cancer,” or “NSCLC.” These terms were arranged into different combinations when used for the search. Besides, the reference lists of original studies were searched manually for additional literature. All the eligible studies were checked carefully to prevent overlapping datasets, and only previously published studies were included.

The studies that fulfilled the following criteria were considered eligible: (1) studies focused on the association between VEGF and the risk of lung cancer; (2) studies used a case–control or cohort design; and (3) inclusion of sufficient data on the frequency of genotypes including ORs and their 95% CIs. In addition, the studies that did not meet the aforementioned inclusion criteria were excluded.

### 2.2. Data Extraction

The data were independently extracted from all the eligible studies by two investigators (Yang FM and Qin ZQ) according to the aforementioned inclusion criteria. When it came to conflicting evaluations, an agreement was settled after discussion with the third reviewer. For each study, the extracted information included: the surname of the first author, year of publication, ethnicity, source of controls, sample size of case and control groups, SNP genotyping methods, genotype distributions, and the results of the Hardy–Weinberg equilibrium (HWE) test.

### 2.3. Statistical Analysis

The pooled odds ratios (ORs) and their corresponding 95% CIs were calculated to evaluate the strength of association between VEGF gene polymorphisms and lung cancer risk under five genetic models: allele model, homozygous model, heterozygous model, dominant model, and recessive model. The genotypic distributions of different polymorphic sites in the subjects were compared with those expected under HWE using the chi-squared goodness-of-fit test, and a* P* value <0.05 was considered to indicate statistically significant heterogeneity.

The pooled ORs were calculated using the fixed-effects model or the random-effects model according to the presence (*P* < 0.05) or absence (*P* > 0.05) of heterogeneity. When the* P *value was >0.05, the pooled ORs were calculated using the fixed-effects model based on the Mantel–Haenszel method. Otherwise, the random-effects model with the DerSimonian–Laird method was used for this meta-analysis. Then, the sources of heterogeneity were further analyzed according to ethnicity. The sensitivity analysis was performed by sequentially excluding individual studies to evaluate the stability and reliability of the results. In addition, publication bias between the studies was analyzed using Begg's funnel plots and Egger's linear regression tests. All the aforementioned statistical tests were performed using Stata12.0 software.

## 3. Results

### 3.1. Studies Characteristics

**Figure 1:**
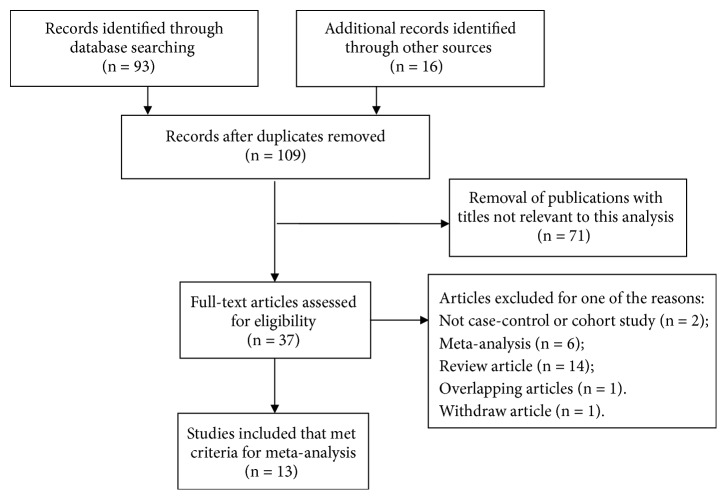
The flowchart of literature search and selection procedure.

According to the searching criteria, a total of 109 studies were initially identified through a primary search of PubMed, Embase, and Web of Science databases and reference lists. Among these studies, 13 full-text studies met the inclusion criteria and were included in the present meta-analysis for further evaluation, which were accrued between December 1992 and July 2012 [27–39]. Besides, all studies suggested that the distribution of genotypes in the controls was consistent under HWE. The flowchart of literature search and selection procedure is shown in Figure 1. The baseline characteristics of the studies associated with the risk of lung cancer are comprehensively listed in Tables 1 and 2. Among these 13 enrolled studies, 10 were based on Asian populations and 3 on Caucasian populations.

### 3.2. Quantitative Synthesis Results

Overall, the strength of association between VEGF genetic polymorphisms and lung cancer risk was evaluated using the pooled ORs with 95% CIs based on five genetic comparison models. A summary of all the meta-analysis results for the 13 studied VEGF polymorphisms and lung cancer susceptibility is provided in Table 3.

### 3.3. +936C/T and Lung Cancer Risk

**Figure 2:**
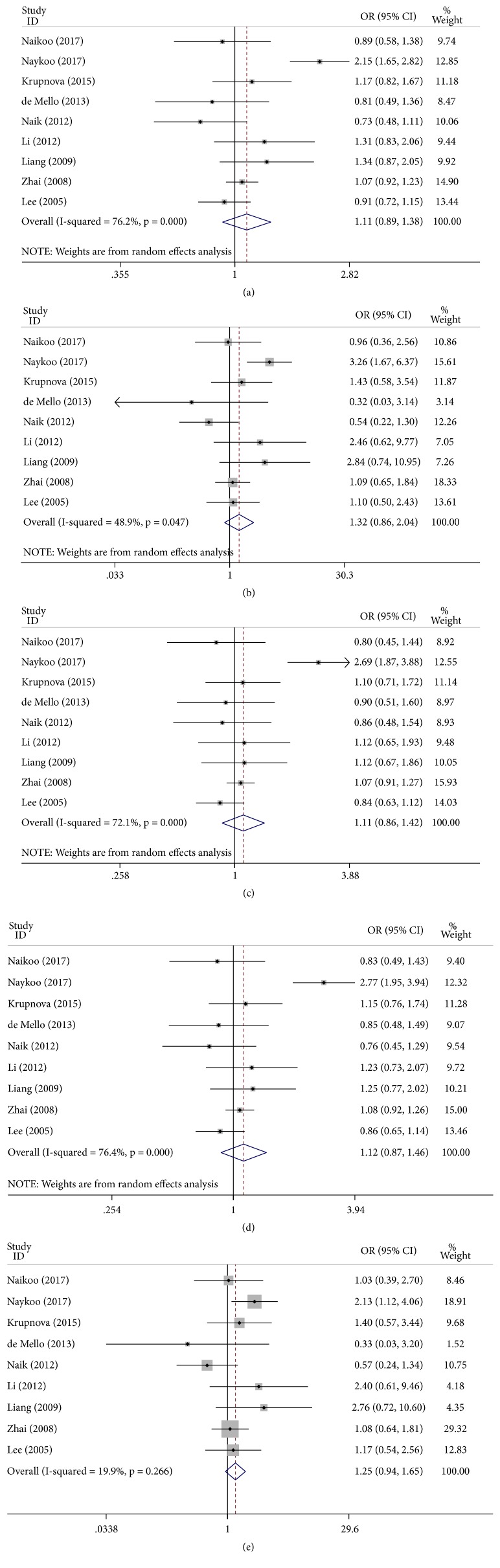
Forest plot of the association between the +936C/T polymorphism and lung cancer risk. (a) Allele model; (b) homozygote model; (c) heterozygote model; (d) dominant model; (e) recessive model.

In the present meta-analysis, the combined results of all analyses showed that the pooled OR of nine studies was 1.11 (95% CI: 0.89–1.38,* P* < 0.001) for the allele model, 1.32 (95% CI: 0.86–2.04,* P* = 0.047) for the homozygote model, 1.11 (95% CI: 0.86–1.42,* P* < 0.001) for the heterozygote model, 1.12 (95% CI: 0.87–1.46,* P* < 0.001) for the dominant model, and 1.25 (95% CI: 0.94–1.65,* P* = 0.266) for the recessive model, indicating no association between VEGF +936C/T mutation and lung cancer susceptibility (Figure 2). Furthermore, subgroup analyses by ethnicity and source of control were performed to establish the effects of heterogeneity on the results. In the subgroup analyses by ethnicity and source of control, no significant results were found in all genetic comparison models (Table 3).

### 3.4. -460C/T and Lung Cancer Risk

**Figure 3:**
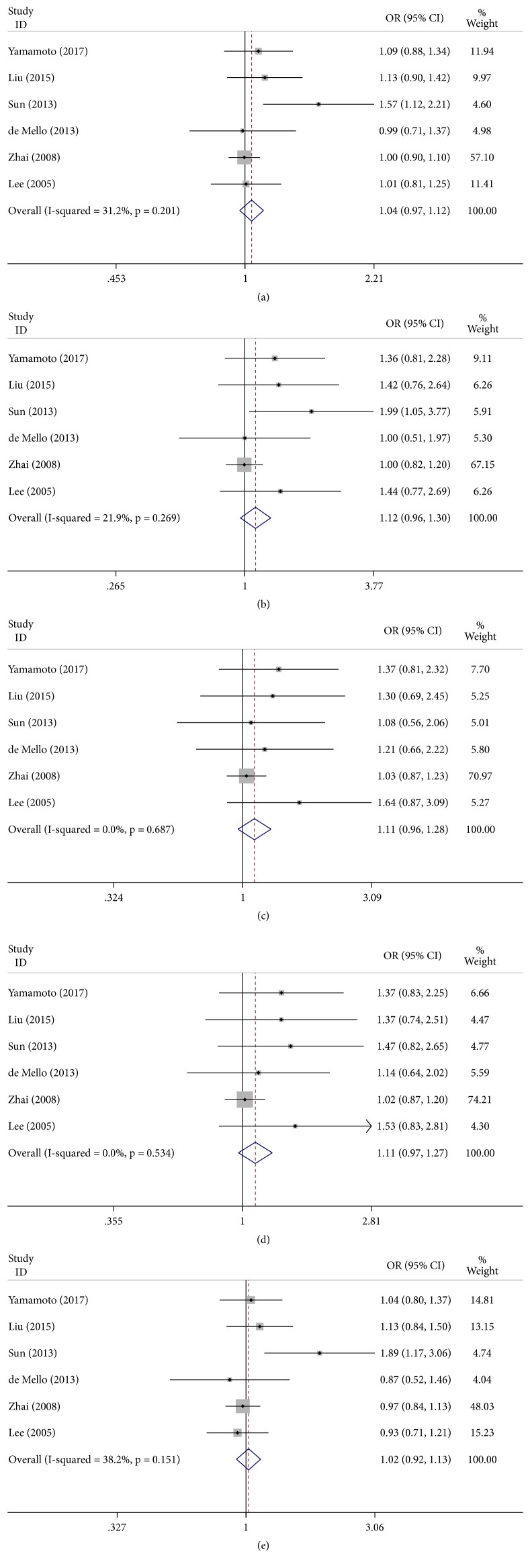
Forest plot of the association between the -460C/T polymorphism and lung cancer risk. (a) Allele model; (b) homozygote model; (c) heterozygote model; (d) dominant model; (e) recessive model.

The results demonstrated that the VEGF -460C/T polymorphism was not significantly correlated with the risk of lung cancer only in the allele model (pooled OR = 1.04, 95% CI: 0.97–1.12,* P* = 0.201), homozygote model (pooled OR = 1.12, 95% CI: 0.96–1.30,* P* = 0.269), heterozygote model (pooled OR = 1.11, 95% CI: 0.96–1.28,* P* = 0.687), dominant model (pooled OR = 1.11, 95% CI: 0.97–1.27,* P* = 0.534), and recessive model (pooled OR = 1.02, 95% CI: 0.92–1.13,* P* = 0.151) (Figure 3). When the studies were stratified by ethnicity, significant differences were observed in Asian populations in these models (allele model: pooled OR = 1.12, 95% CI: 1.00–1.26; homozygote model: pooled OR = 1.51, 95% CI: 1.12–2.03), but no significant results were detected in Caucasian populations. Furthermore, significant results were found in the homozygote model (pooled OR = 1.59, 95% CI: 1.11–2.29) in the subgroup analysis by the source of control (Table 3).

### 3.5. -2578C/A and Lung Cancer Risk

**Figure 4:**
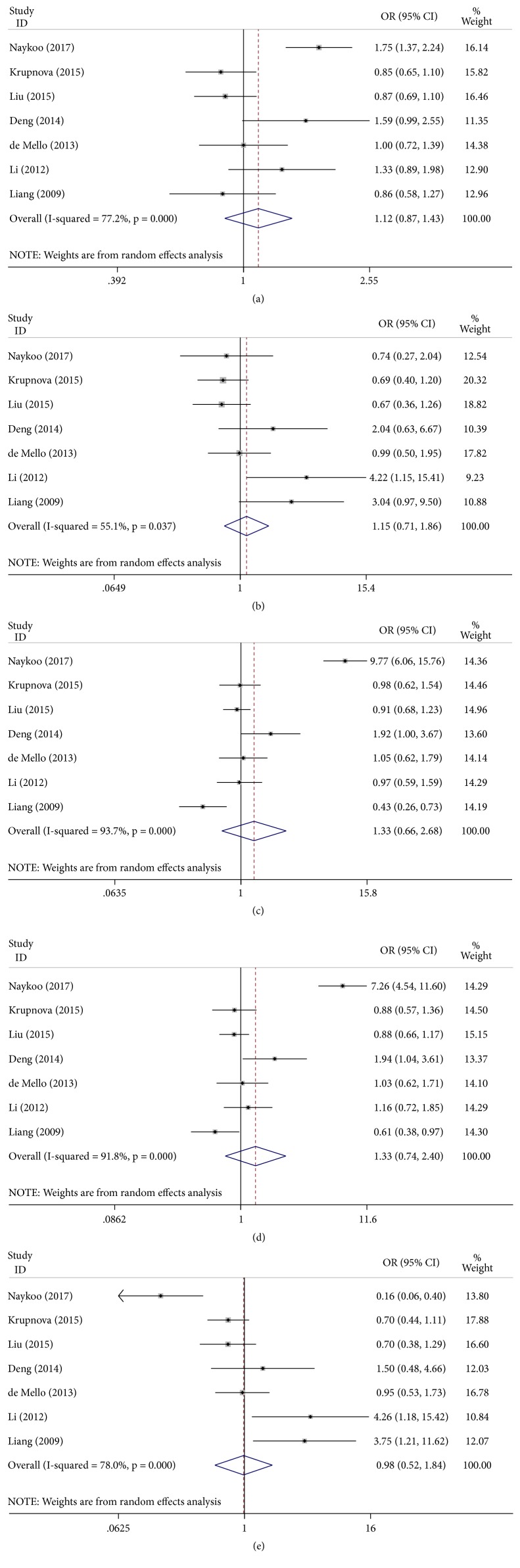
Forest plot of the association between the -2578C/A polymorphism and lung cancer risk. (a) Allele model; (b) homozygote model; (c) heterozygote model; (d) dominant model; (e) recessive model.

The combined results of all analyses showed that the pooled OR of these studies was 1.12 (95% CI: 0.87–1.43,* P* < 0.001) for the allele model, 1.15 (95% CI: 0.71–1.86,* P* = 0.037) for the homozygote model, 1.33 (95% CI: 0.66–2.68,* P* < 0.001) for the heterozygote model, 1.33 (95% CI: 0.74–2.40,* P* < 0.001) for the dominant model, and 0.98 (95% CI: 0.52–1.84,* P* < 0.001) for the recessive model, indicating no significant association between VEGF -2578C/A polymorphism and lung cancer risk (Figure 4). In addition, when the studies were stratified by ethnicity and source of control, no significant differences were found in all genetic models (Table 3).

### 3.6. Sensitivity Analysis

**Figure 5:**
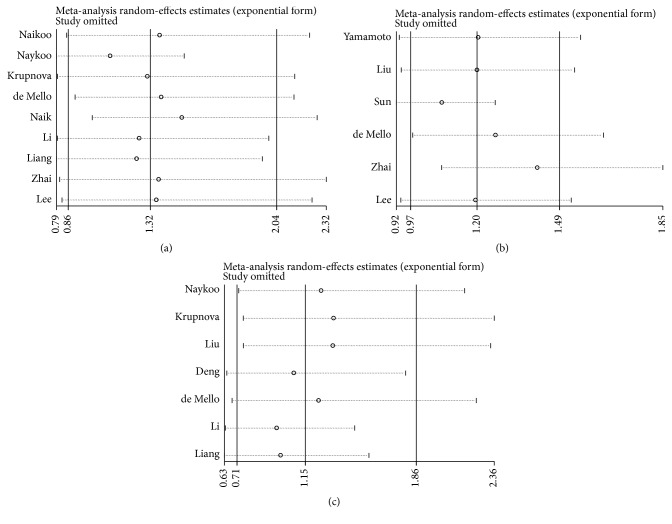
Sensitivity analysis in the homozygote model. (a) +936C/T polymorphism; (b) -460C/T polymorphism; (c) -2578C/A polymorphism.

Individual studies were consecutively omitted in the sensitivity analysis to detect the influence of each study on the pooled OR. The sensitivity analysis for the results of VEGF genetic polymorphisms and lung cancer risk demonstrated that the obtained results were statistically robust, and no individual study affected the pooled OR significantly (Figure 5).

### 3.7. Publication Bias

**Figure 6:**
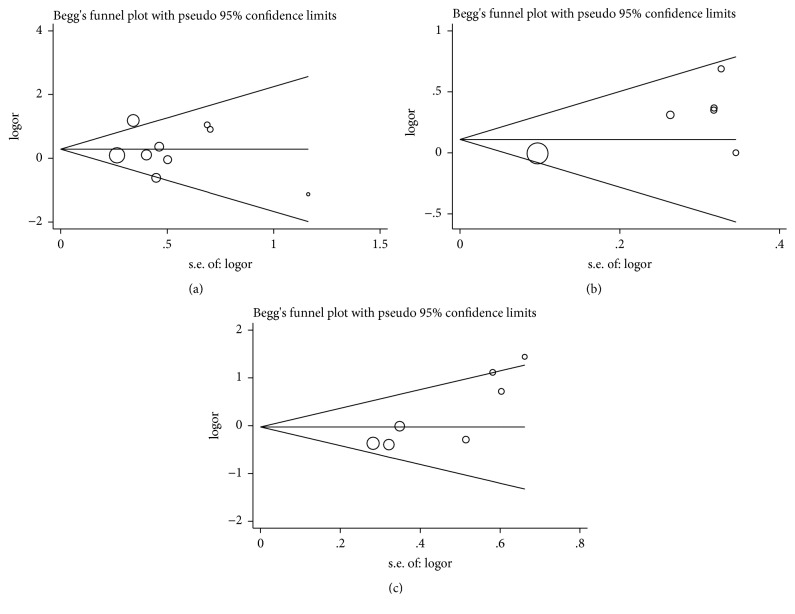
Begg's funnel plot of publication bias test in the homozygote model. (a) +936C/T polymorphism; (b) -460C/T polymorphism; (c) -2578C/A polymorphism.

The possible publication bias of the studies involved in the present meta-analysis was examined via Begg's funnel plot and Egger's test. As shown in Figure 6, the shapes of funnel plots showed no evidence of publication bias in the dominant model (+936C/T,* P* = 0.754; -460C/T,* P* = 0.133; -2578C/A,* P* = 0.230). Result from Begg's test and Egger's test also confirmed the absence of publication bias. Considering the above tests, the results of our meta-analysis were reliable.

## 4. Discussion

As an important regulator of various malignancy angiogenesis, VEGF is always involved in the development and progression of multiple cancers [25]. The process of carcinogenesis is accelerated when the VEGF gene expression is influenced by some molecular biology variations [40]. Previous studies have identified at least 30 SNPs of VEGF [18]. Some of these have been confirmed to be associated with individual susceptibility to various types of cancers, including lung cancer [41, 42]. In the last two decades, many case–control studies have assessed the association of VEGF polymorphisms with the risk of lung cancer. However, due to the limited sample size and other reasons, the findings are controversial or ambiguous [27–39]. In addition, a recent meta-analysis suggested that VEGF -2578C/A polymorphism was capable of increasing the susceptibility to lung cancer, especially among smokers and patients with lung squamous cell carcinoma. Additionally, for +936C/T polymorphism, increased lung cancer susceptibility was observed only among patients with lung adenocarcinoma. In contrast, VEGF -460C/T polymorphism might be a protective factor among nonsmokers and patients with SCC [43]. However, several other studies showed that the -460C/T and -2578C/A polymorphisms of VEGF were not associated with an increased risk of lung cancer [44–46]. The conclusion in previous studies was still inaccurate due to limited published data and lack of subgroup analysis. Therefore, this updated meta-analysis including all eligible case–control studies was performed to investigate these associations.

This meta-analysis included 13 independent case–control studies with a total of 4477 patients with lung cancer and 4346 healthy controls to investigate the correlation between VEGF variants and the risk of lung cancer. The results of our meta-analysis showed that VEGF +936C/T, -460C/T, and -2578C/A gene polymorphisms were not associated with the risk of lung cancer. However, the conclusion was still inaccurate due to limited published data. Furthermore, the predictive value of VEGF polymorphisms in the prognosis of lung cancer could not be assessed. The sample size in the present meta-analysis was larger than that in any individual study, leading to more precise and robust results. Thus, the present meta-analysis aimed to provide a more powerful and reliable conclusion about the relationship between VEGF genetic polymorphisms and lung cancer risk. Moreover, no statistically significant association was detected between VEGF +936C/T, -460C/T, and -2578C/A polymorphisms and lung cancer risk in all genetic comparison models. Interestingly, the VEGF +460T/C polymorphism was found to be significantly associated with the susceptibility to lung cancer only in Asian populations. Although the exact mechanism underlying ethnic differences was unknown, the possible reasons could be the natural selection pressures or a balance by other related functional genetic variants and environmental exposures, resulting in gene polymorphisms [47]. Each polymorphism alone may be insufficient to influence the susceptibility to lung cancer.

However, the present meta-analysis had several limitations. First, the number of included studies in our meta-analysis was not so abundant, leading to limited statistical precision, especially in subgroup analysis. Hence, more studies using standardized unbiased methods are needed to offer more detailed individual data. Second, the population included in this meta-analysis is mainly limited to Asian and Caucasian ethnicities, and more data from other ethnic groups will be required. Third, since only data from previously published studies were included in the meta-analysis, it was possible that some related published studies or unpublished studies had null results, resulting in a deviation from the expected results. Finally, tumorigenesis is a complex process involved in the regulation of a series of genetic factors besides VEGF. As a multifactorial disease, the risk of developing lung cancer was closely related to the environment, smoking, occupational exposure, and interaction among various genetic factors, and not just a single factor. Therefore, more raw data are needed to adjust other variables, such as age, sex, and smoking. Consequently, further high-quality studies on the risk of lung cancer should be conducted over the next few years to achieve more accurate results.

## 5. Conclusions

In sum, this meta-analysis found that VEGF +936C/T, -460C/T, and -2578C/A polymorphisms were not associated with the susceptibility to lung cancer. Interestingly, the VEGF +460T/C polymorphism might be a risk factor for lung cancer only in Asian populations. Based on our discovery, additional large population-based multicenter prospective studies are needed to confirm the association of VEGF** gene polymorphisms** and lung cancer risk.

## Figures and Tables

**Table 1:** Baseline characteristics of studies associated with the risk of lung cancer included in the meta-analysis.

Year	Surname	Ethnicity	Country	SOC	Genotyping	Cases	Controls	Gene polymorphism
2017	Yamamoto	Japan	Asian	HB	TaqMan	462	379	-460C/T
2017	Naikoo	India	Asian	PB	PCR-RFLP	112	116	+936C/T
2017	Naykoo	India	Asian	PB	PCR-RFLP	199	401	+936C/T, -2578C/A
2015	Krupnova	Belarus	Caucasian	HB	PCR-RFLP	202	336	+936C/T, -2578C/A
2015	Liu	China	Asian	PB	PCR-RFLP	414	338	-460C/T, -2578C/A
2014	Deng	China	Asian	HB	PCR-RFLP	65	110	-2578C/A
2013	Sun	China	Asian	PB	PCR-RFLP	126	160	-460C/T
2013	de Mello	Portugal	Caucasian	HB	MassARRAY	144	144	+936C/T, -460C/T, -2578C/A
2012	Naik	India	Asian	PB	PCR-RFLP	100	150	+936C/T
2012	Li	China	Asian	PB	PCR-RFLP	150	150	+936C/T, -2578C/A
2009	Liang	China	Asian	PB	PCR-RFLP	171	172	+936C/T, -2578C/A
2008	Zhai	America	Caucasian	HB	TaqMan	1900	1458	+936C/T, -460C/T
2005	Lee	Korea	Asian	PB	PCR-RFLP	432	432	+936C/T, -460C/T

SOC: source of control; HB: hospital-based; PB: population-based controls.

**Table 2:** The genotype frequency data of individual studies included in the meta-analysis.

VEGF	rs3025039	+936C/T			Case (n)			Control (n)		

Year	Surname	Case	Control	CC	CT	TT	CC	CT	TT	HWE

2017	Naikoo	109	112	68	32	9	65	38	9	Y
2017	Naykoo	199	401	80	99	20	261	120	20	Y
2015	Krupnova	161	360	115	38	8	267	80	13	N
2013	de Mello	144	144	115	28	1	111	30	3	Y
2012	Naik	90	150	54	28	8	80	48	22	N
2012	Li	150	150	108	35	7	114	33	3	Y
2009	Liang	171	172	123	40	8	131	38	3	Y
2008	Zhai	1900	1458	1441	424	35	1125	308	25	Y
2005	Lee	432	432	281	137	14	266	154	12	Y

VEGF	rs833061	-460C/T								

2017	Yamamoto	462	379	32	197	233	35	157	187	Y
2015	Liu	414	338	21	164	229	23	138	177	Y
2013	Sun	126	160	22	43	61	38	69	53	Y
2013	de Mello	144	144	28	79	37	31	72	41	Y
2008	Zhai	1900	1458	439	922	539	342	694	422	Y
2005	Lee	430	432	18	184	228	27	168	237	Y

VEGF	rs699947	-2578C/A								

2017	Naykoo	199	401	24	170	5	200	145	56	N
2015	Krupnova	162	360	41	90	31	83	186	91	Y
2015	Liu	414	338	230	164	20	177	138	23	Y
2014	Deng	65	110	26	33	6	62	41	7	Y
2013	de Mello	144	144	43	75	26	44	73	27	Y
2012	Li	150	150	93	45	12	98	49	3	Y
2009	Liang	171	172	129	28	14	112	56	4	Y

**Table 3:** Meta-analysis results for the included studies of the association between rs3025039, rs833061, and rs699947 polymorphisms and risk of lung cancer.

Variables	No. of studies	Dominant model			Recessive model			Homozygous model			Heterozygous model			Allele model		

		OR (95% CI)	P values	I-squared (%)	OR (95% CI)	P values	I-squared (%)	OR (95% CI)	P values	I-squared (%)	OR (95% CI)	P values	I-squared (%)	OR (95% CI)	P values	I-squared (%)

rs3025039 C>T	+936C/T	(CT + TT) vs. CC			TT vs. (CT + CC)			TT vs. CC			CT vs. CC			T vs. C		
All	9	1.12 (0.87-1.46)	<0.001	76.4	1.25 (0.94-1.65)	0.266	19.9	1.32(0.86-2.04)	0.047	48.9	1.11 (0.86-1.42)	<0.001	72.1	1.11 (0.89-1.38)	<0.001	76.2
Ethnicity																
Asian	6	1.17 (0.75-1.81)	<0.001	84.4	1.37 (0.95-1.97)	0.154	37.9	1.47 (0.78-2.77)	0.022	61.9	1.14 (0.74-1.75)	<0.001	82	1.15(0.81-1.65)	<0.001	83.8
Caucasian	3	1.07 (0.92-1.23)	0.681	0	1.10 (0.70-1.70)	0.507	0	1.11 (0.71-1.73)	0.488	0	1.07 (0.92-1.24)	0.836	0	1.06 (0.93-1.21)	0.522	0
Source of control																
HB	3	1.07 (0.92-1.23)	0.681	0	1.10 (0.70-1.70)	0.507	0	1.11 (0.71-1.73)	0.488	0	1.07(0.92-1.24)	0.836	0	1.06 (0.93-1.21)	0.522	0
PB	6	1.17 (0.75-1.81)	<0.001	84.4	1.37 (0.95-1.97)	0.154	37.9	1.47 (0.78-2.77)	0.022	61.9	1.14 (0.74-1.75)	<0.001	82	1.15(0.81-1.65)	<0.001	83.8
rs833061 C>T	-460C/T	(CT + TT) vs. CC			TT vs. (CT + CC)			TT vs. CC			CT vs. CC			T vs. C		
All	6	1.11(0.97-1.27)	0.534	0	1.02(0.92-1.13)	0.151	38.2	1.12 (0.96-1.30)	0.269	21.9	1.11 (0.96-1.28)	0.687	0	1.04 (0.97-1.12)	0.201	31.2
Ethnicity																
Asian	4	1.42 (1.07-1.90)	0.991	0	1.09 (0.94-1.27)	0.085	54.6	**1.51 (1.12-2.03)**	0.821	0	1.34 (0.99-1.81)	0.837	0	**1.12 (1.00-1.26)**	0.184	38
Caucasian	2	1.03 (0.88-1.20)	0.721	0	0.96 (0.83-1.11)	0.684	0	1.00 (0.83-1.20)	0.991	0	1.05 (0.89-1.24)	0.616	0	0.99 (0.91-1.09)	0.957	0
Source of control																
HB	3	1.05 (0.91-1.22)	0.531	0	0.98 (0.86-1.11)	0.807	0	1.03 (0.87-1.23)	0.532	0	1.07 (0.92-1.26)	0.554	0	1.01 (0.93-1.10)	0.757	0
PB	3	**1.45 (1.03-2.06)**	0.968	0	1.11 (0.93-1.33)	0.039	69.1	**1.59 (1.11-2.29)**	0.708	0	1.33 (0.92-1.91)	0.656	0	1.14 (0.99-1.31)	0.095	57.5
rsrs699947 C>A	-2578C/A	(CA + AA) vs. CC			AA vs. (AA + CC)			AA vs. CC			AA vs. CC			A vs. C		
All	7	1.33(0.74-2.40)	<0.001	91.8	0.98(0.52-1.84)	<0.001	78	1.15(0.71-1.86)	0.037	55.1	1.33 (0.66-2.68)	<0.001	93.7	1.12 (0.87-1.43)	<0.001	77.2
Ethnicity																
Asian	5	1.53 (0.67-3.51)	<0.001	94.3	1.15(0.37-3.52)	<0.001	84.9	1.50(0.71-3.20)	0.026	63.9	1.48(0.54-4.04)	<0.001	95.7	1.22 (0.87-1.70)	<0.001	80.6
Caucasian	2	0.94(0.68-1.31)	0.654	0	0.79(0.55-1.13)	0.417	0	0.79 (0.52-1.22)	0.426	0	1.01(0.72-1.42)	0.842	0	0.90 (0.74-1.11)	0.434	0
Source of control																
HB	3	1.15 (0.75-1.77)	0.122	52.6	0.83 (0.59-1.18)	0.411	0	0.93 (0.56-1.54)	0.247	28.6	1.19(0.82-1.73)	0.224	33.2	1.05 (0.76-1.45)	0.073	61.8
PB	4	1.45(0.54-3.90)	<0.001	95.6	1.09 (0.27-4.34)	<0.001	88.2	1.43 (0.58-3.54)	0.017	70.4	1.39 (0.42-4.65)	<0.001	96.7	1.15 (0.79-1.69)	<0.001	84.3

## References

[1] Siegel R. L., Miller K. D., Jemal A. (2016). Cancer statistics, 2016. *CA: A Cancer Journal for Clinicians*.

[2] Torre L. A., Siegel R. L., Jemal A. (2016). Lung cancer statistics. *Advances in Experimental Medicine and Biology*.

[3] Hensing T., Chawla A., Batra R., Salgia R. (2014). A personalized treatment for lung cancer: Molecular pathways, targeted therapies, and genomic characterization. *Advances in Experimental Medicine and Biology*.

[4] Jahangeer S., Forde P., Soden D., Hinchion J. (2013). Review of current thermal ablation treatment for lung cancer and the potential of electrochemotherapy as a means for treatment of lung tumours. *Cancer Treatment Reviews*.

[5] Chen K., Zhou Y.-X., Li K. (2016). A novel three-round multiplex PCR for SNP genotyping with next generation sequencing. *Analytical and Bioanalytical Chemistry*.

[6] Mousa S. A., Lin H.-Y., Tang H. Y., Hercbergs A., Luidens M. K., Davis P. J. (2014). Modulation of angiogenesis by thyroid hormone and hormone analogues: implications for cancer management. *Angiogenesis*.

[7] Na H.-J., Hwang J.-Y., Lee K.-S. (2014). TRAIL negatively regulates VEGF-induced angiogenesis via caspase-8-mediated enzymatic and non-enzymatic functions. *Angiogenesis*.

[8] Ahluwalia A., Jones M. K., Matysiak-Budnik T., Tarnawski A. S. (2014). VEGF and colon cancer growth beyond angiogenesis: Does VEGF directly mediate colon cancer growth via a non-angiogenic mechanism?. *Current Pharmaceutical Design*.

[9] Matsumoto K., Ema M. (2014). Roles of VEGF-A signalling in development, regeneration, and tumours. *The Journal of Biochemistry*.

[10] Rezzola S., Belleri M., Gariano G. (2014). In vitro and ex vivo retina angiogenesis assays. *Angiogenesis*.

[11] Fu B. H., Fu Z. Z., Meng W., Gu T., Sun X. D., Zhang Z. (2015). Platelet VEGF and serum TGF-*β*1 levels predict chemotherapy response in non-small cell lung cancer patients. *Tumor Biology*.

[12] Fu Z.-Z., Sun X.-D., Li P. (2014). Relationship between serum VEGF level and radiosensitivity of patients with nonsmall cell lung cancer among asians: A meta-analysis. *DNA and Cell Biology*.

[13] Alevizakos M., Kaltsas S., Syrigos K. N. (2013). The VEGF pathway in lung cancer. *Cancer Chemotherapy and Pharmacology*.

[14] Hicklin D. J., Ellis L. M. (2005). Role of the vascular endothelial growth factor pathway in tumor growth and angiogenesis. *Journal of Clinical Oncology*.

[15] Mountzios G., Pentheroudakis G., Carmeliet P. (2014). Bevacizumab and micrometastases: Revisiting the preclinical and clinical rollercoaster. *Pharmacology & Therapeutics*.

[16] Lauro S., Onesti C. E., Righini R., Marchetti P. (2014). The use of bevacizumab in non-small cell lung cancer: An update. *Anticancer Reseach*.

[17] Giacca M., Zacchigna S. (2012). VEGF gene therapy: Therapeutic angiogenesis in the clinic and beyond. *Gene Therapy*.

[18] Ruggiero D., Dalmasso C., Nutile T. (2011). Genetics of VEGF serum variation in human isolated populations of Cilento: Importance of VEGF polymorphisms. *PLoS ONE*.

[19] Hein A., Lambrechts D., Von Minckwitz G. (2015). Genetic variants in VEGF pathway genes in neoadjuvant breast cancer patients receiving bevacizumab: Results from the randomized phase III GeparQuinto study. *International Journal of Cancer*.

[20] Tie Z., Bai R., Zhai Z. (2014). Single nucleotide polymorphisms in VEGF gene are associated with an increased risk of osteosarcoma. *International Journal of Clinical and Experimental Pathology*.

[21] Renner W., Kotschan S., Hoffmann C., Obermayer-Pietsch B., Pilger E. (2000). A common 936 C/T mutation in the gene for vascular endothelial growth factor is associated with vascular endothelial growth factor plasma levels. *Journal of Vascular Research*.

[22] Koukourakis M. I., Papazoglou D., Giatromanolaki A., Bougioukas G., Maltezos E., Siviridis E. (2004). VEGF gene sequence variation defines VEGF gene expression status and angiogenic activity in non-small cell lung cancer. *Lung Cancer*.

[23] Yin M., Liao Z., Yuan X. (2012). Polymorphisms of the vascular endothelial growth factor gene and severe radiation pneumonitis in non-small cell lung cancer patients treated with definitive radiotherapy. *Cancer Science*.

[24] Dong J., Dai J., Shu Y. (2010). Polymorphisms in EGFR and VEGF contribute to non-small-cell lung cancer survival in a Chinese population. *Carcinogenesis*.

[25] Ferrara N., Gerber H. P., LeCouter J. (2003). The biology of VEGF and its receptors. *Nature Medicine*.

[26] Naykoo N. A., Hameed I., Aasif M. (2013). WITHDRAWN: Single nucleotide polymorphisms, haplotype association and tumour expression of the vascular endothelial growth factor (VEGF) gene with lung carcinoma. *Gene*.

[27] Yamamoto Y., Kiyohara C., Ogata-Suetsugu S., Hamada N., Nakanishi Y. (2017). Association between genetic polymorphisms involved in the hypoxia-inducible factor pathway and lung cancer risk: a case–control study in Japan. *Asia-Pacific Journal of Clinical Oncology*.

[28] Naykoo N. A., Dil-Afroze, Rasool R. (2017). Single nucleotide polymorphisms, haplotype association and tumour expression of the vascular endothelial growth factor (VEGF) gene with lung carcinoma. *Gene*.

[29] Naikoo N. A., Afroze D., Rasool R. (2017). SNP and haplotype analysis of vascular endothelial growth factor (VEGF) gene in lung cancer patients of Kashmir. *Asian Pacific Journal of Cancer Prevention*.

[30] Liu C., Zhou X., Gao F., Qi Z., Zhang Z., Guo Y. (2015). Correlation of genetic polymorphism of vascular endothelial growth factor gene with susceptibility to lung cancer. *Cancer Gene Therapy*.

[31] Krupnova E. V., Shapetska M. N., Mikhalenko E. P. (2015). Role of vascular endothelial growth factor in non-small cell lung cancer pathogenesis. *Experimental Oncology*.

[32] Deng Z.-C., Cao C., Yu Y.-M., Ma H.-Y., Ye M. (2014). Vascular endothelial growth factor -634G/C and vascular endothelial growth factor -2578C/A polymorphisms and lung cancer risk: A case-control study and meta-analysis. *Tumor Biology*.

[33] Sun S.-F., Huang D.-B., Cao C., Deng Z.-C. (2013). Polymorphism of VEGF-460C/T associated with the risk and clinical characteristics of lung cancer in Chinese population. *Medical Oncology*.

[34] de Mello R. A., Ferreira M., Soares-Pires F. (2013). The Impact of Polymorphic Variations in the 5p15, 6p12, 6p21 and 15q25 Loci on the Risk and Prognosis of Portuguese Patients with Non-Small Cell Lung Cancer. *PLoS ONE*.

[35] Li Y., Liang J., Liu X. (2012). Correlation of polymorphisms of the vascular endothelial growth factor gene and the risk of lung cancer in an ethnic han group of North China. *Experimental and Therapeutic Medicine*.

[36] Naik N. A., Bhat I. A., Afroze D. (2012). Vascular endothelial growth factor A gene (VEGFA) polymorphisms and expression of VEGFA gene in lung cancer patients of Kashmir Valley (India). *Tumor Biology*.

[37] Liang J., Yu X., Liu X. (2009). Vascular endothelial growth factor polymorphisms and risk of lung cancer. *The Chinese-German Journal of Clinical Oncology*.

[38] Zhai R., Liu G., Zhou W. (2008). Vascular endothelial growth factor genotypes, haplotypes, gender, and the risk of non-small cell lung cancer. *Clinical Cancer Research*.

[39] Lee S. J., Lee S. Y., Jeon H.-S. (2005). Vascular endothelial growth factor gene polymorphisms and risk of primary lung cancer. *Cancer Epidemiology, Biomarkers & Prevention*.

[40] Arcondeguy T., Lacazette E., Millevoi S., Prats H., Touriol C. (2013). VEGF-A mRNA processing, stability and translation: a paradigm for intricate regulation of gene expression at the post-transcriptional level. *Nucleic Acids Research*.

[41] Jannuzzi A. T., Özhan G., Yanar H. T., Alpertunga B. (2015). VEGF gene polymorphisms and susceptibility to colorectal cancer. *Genetic Testing and Molecular Biomarkers*.

[42] Chen G.-Q., Luo J.-B., Wang G.-Z., Ding J.-E. (2014). Assessment of the associations between three VEGF polymorphisms and risk of prostate cancer. *Tumor Biology*.

[43] Lin L., Cao K., Chen W., Pan X., Zhao H. (2013). Four common vascular endothelial growth factor polymorphisms (-2578C>A, -460C>T, +936C>T, and +405G>C) in susceptibility to lung cancer: a meta-analysis. *PLoS ONE*.

[44] Tu J., Wang S., Zhao J. (2014). rs833061 and rs699947 on Promoter Gene of Vascular Endothelial Growth Factor (VEGF) and Associated Lung Cancer Susceptibility and Survival: A Meta-Analysis. *Medical Science Monitor*.

[45] Yu W., Jiang X., Bai T., Lv X., Chang F. (2014). Association between +936 C>T gene polymorphism of vascular endothelial growth factor and lung cancer: A meta-analysis. *Cancer Biomarkers*.

[46] Chen Q., Zhou Z., Shan L. (2014). Association of the vascular endothelial growth factor -2578C/A polymorphism with cancer risk: a meta-analysis update. *Biomedical Reports*.

[47] Stevens A., Soden J., Brenchley P. E., Ralph S., Ray D. W. (2003). Haplotype analysis of the polymorphic human vascular endothelial growth factor gene promoter. *Cancer Research*.

